# Diet Quality Index and Food Choice Motives in Vietnam: The Roles of Sensory Appeal, Mood, Convenience, and Familiarity

**DOI:** 10.3390/foods12132505

**Published:** 2023-06-28

**Authors:** Huong Thi Trinh, Binh Thi Thanh Dao, Tuyen Thi Thanh Huynh, Mai Thi Tuyet Nguyen, Trang Mai Nguyen, Vy Thao Vuong, Thanh Thi Duong, Stef de Haan

**Affiliations:** 1 Faculty of Mathematical Economics, Thuongmai University, Hanoi 100000, Vietnam; tuyetmainguyen@tmu.edu.vn; 2 Faculty of Management and Tourism, Hanoi University, Hanoi 100000, Vietnam; 3 International Center for Tropical Agriculture (CIAT)-Asia Office, Hanoi 100000, Vietnam; t.huynh@cgiar.org (T.T.T.H.); t.t.duong@cgiar.org (T.T.D.); 4 Wageningen Economic Research, Wageningen University and Research, 6708 WB Wageningen, The Netherlands; trang.nguyen@wur.nl; 5 Department of Global Development, College of Agriculture and Life Sciences, Cornell University, Ithaca, NY 14853, USA; vtv6@cornell.edu; 6 International Potato Center (CIP), Avenida La Molina 1895, Apartado 1558, Lima 15023, Peru; s.dehaan@cgiar.org

**Keywords:** Vietnam, food choice questionnaire, diet quality index, rural–urban transect, explanatory factor analysis, structural equation modeling

## Abstract

Food choices that shape human diets and health are influenced by various socio-economic factors. Vietnam struggles to meet many nutrition targets where links between food choice and diet have not been widely explored. This study assesses the food choice motives, based on a 28-item food choice questionnaire (FCQ), and the diet quality of 603 adults in three sites (urban, peri-urban, and rural) in northern Vietnam. We assess diet quality using the Diet Quality Index–Vietnam (DQI-V) which consists of variety, adequacy, moderation, and balance components. Using factor analysis, we grouped FCQ items into five factors: health focus, sensory appeal, mood ethics, convenience, and familiarity. The structural equation modeling indicates that food choice motives significantly impact the DQI-V and its components but in different directions. The results show that sensory appeal has a positive association with the overall DQI-V score, while having a negative impact on the variety component. Findings present a potential trade-off issue for interventions and policies related to food products. Nutrition knowledge is positively associated with all elements of diet quality across all three study sites. Vietnamese agrobiodiversity could be better utilized to increase dietary diversity. Differentiated policies are necessary to address the poor dietary diversity and adequacy in northern Vietnam.

## 1. Introduction

Studying food choices is crucial for understanding food choices and identifying consumer demand for certain foods, thereby providing potentially important insight into dietary and health outcomes. Food choices have been utilized to explain sustainable consumption [1], personalized nutrition adoption [2], and differences in consumption due to the global pandemic [3]. Adequate food choices have immediate and long-lasting effects on the physical and mental health of consumers and their future well-being [4].

The food choice questionnaire (FCQ) is a widely used instrument to capture the consumers’ perceptions, motives, and attitudes toward food. It has been designed, tested, and validated in more than 40 countries in varied contexts [5]. The FCQ has been a useful tool to identify socio-economic factors that are connected to food choices and health implications. For example, Marsola et al. [6] recently used the FCQ to define clusters of Brazilian consumers according to risk perceptions about chronic diseases. The FCQ has also been used in conjunction with dietary or food behavioral measures to uncover patterns and associations between food motives and consumption outcomes. For instance, Cabral et al. [5] combined the FCQ and food consumption frequency data to determine the dietary patterns of rural and urban populations in Cape Verde. Røed et al. [7] linked parental food choices with infants’ fruit and vegetable intake to find associations between the motive of health and infants’ vegetable intake. Despite its widespread use across different contexts, the FCQ still requires adaptation to accommodate different food cultures under study [8].

Diet quality has been measured by various indices [9]. For example, the Diet Quality Index–International (DQI-I) has been applied, validated, and proved to be a useful measure of diet quality in various contexts [10,11,12]. It combines nutrient and food/food group-based indicators to provide a more comprehensive picture of diet quality than nutrient or food/food groups alone, covering four aspects of a diet: variety, adequacy, moderation, and balance.

The majority of research combining food choice and diet quality has been conducted in developed countries [1,5]. Yet food cultures differ widely between and within countries. We conducted our study in Vietnam, a lower middle-income country rapidly undergoing a nutrition transition. It struggles to meet many diet and nutrition targets. Its food system has been transforming under economic growth, urbanization, and trade liberalization [13,14]. Vietnam’s general dietary pattern has shifted away from consumption of starchy staples, dark-green vegetables, and legumes, towards that of animal proteins, fat/oils, ripe fruits, processed and pre-prepared foods, and beverages [15]. Vietnam faces a double burden of malnutrition, where under-nutrition (underweight, micronutrient deficiencies) co-exists with over-nutrition (overweight, obesity) [15]. The links between food choice and diet have not been widely explored in Vietnam. Thus, more scientific evidence on food choices and their impacts is needed to inform policymakers towards devising better incentive and regulatory systems to promote healthier food choices.

We have applied an FCQ to a sample of Vietnamese consumers across a rural–urban transect to investigate its applicability and scope for improvement. Thus, the objectives of the present study were: (i) to validate the FCQ approach in Vietnam; (ii) to illustrate the use of DQI-V as a new diet indicator for food quality in Vietnam, which has integrated the guidelines from the Vietnam food pyramid for adults in the period 2016–2020 [16]; and (iii) to elucidate the role of food choice motives in determining diet quality.

We make three main contributions to the growing literature on food choices and diet quality in a lower middle-income country. Firstly, this paper provides additional evidence and proof-of-concept for the food choice questionnaire as a research tool in Vietnam. Secondly, we demonstrate the use of a country-specific diet quality measure (DQI-V). Thirdly, we assess the relationship between DQI-V and FCQ.

## 2. Materials and Methods

### 2.1. Data

Data were collected through in-person surveys as part of the CGIAR Research Program (CRP) on Agriculture for Nutrition and Health or A4NH [17]. The survey was conducted within three districts in northern Vietnam to elucidate specific features of local Vietnamese food systems along a rural to urban transect. The transect included a rural site (Moc Chau district in Son La province), a peri-urban site (Dong Anh district in Hanoi), and an urban site (Cau Giay district, also in Hanoi). The population in the rural site has a high ethnic diversity. Their livelihoods and food consumption are mainly based on their agricultural production. The peri-urban site is characterized by rapid urbanization and intensive agriculture along with agricultural interactions with the urban area. The population in this site also has a high percentage of migrants. In contrast, the urban site represents a typical urban space in the capital with mixed retail outlets.

Households were chosen via a three-stage sample survey. Thirty random villages/clusters as primary sampling units (PSUs) were selected, following a probability proportional to size (PPS) procedure. Ten PSUs were then randomly selected from the 30 PSUs for score clusters. Then, based on the rapid enumeration of households from the district health centers, households were randomly selected for the survey. For the consumer behavior component, the household representative (i.e., the person who was mainly responsible for the household food purchase and/or preparation) was interviewed. For the dietary assessment, 24 h recalls were recorded among households that had children under five. Participation in the survey was completely voluntary. The final sample comprised 603 adults, both men and women, across the three sites.

For more details on the sampling frame, how the study was carried out, and how data were managed, see the full report, “Partial Food Systems Baseline Assessment at the Vietnam Benchmark Sites”, https://cgspace.cgiar.org/handle/10568/113122 (accessed on 1 April 2023) [17].

### 2.2. Measures of Food Choice Motives and Socio-Demographic Variables

Food choice questionnaire: This study used a 28-item questionnaire—an adaptation of the FCQ developed by Steptoe et al. [18]—as in a study in Ethiopia [19]. The original FCQ consisted of 36 items which construct 9 latent variables: health, mood, convenience, sensory appeal, natural content, price, weight control, familiarity, and ethical concerns [18]. Respondents were asked to rate the statement, “It is important to me that the food I eat on a typical day…”, for each of the 36 items on a five-point scale, ranging from “not at all important” to “very important”. The modified questionnaire added some items related to agrochemical use, media/advertising influence, and local people’s perceptions of local concerns. We combined several options: e.g., “takes no time to prepare” with “easy to prepare”; “keeps me healthy”, “helps me control my weight”, and “is good for my skin/teeth/hair/nails/etc.” into one single item, “keeps me healthy (including controlling my weight, good for my skin/teeth/hair/nails, etc.… The options “is cheap” and “is good value for money” were collapsed into one single item, “Is not expensive/cheap/good value for money”. Items “helps me relax”, “keeps me awake/alert”, “cheers me up”, “makes me feel good”, were combined into one item (generic good feelings). Item “is nutritious” was excluded, as it was confused with “providing minerals and vitamins” in Vietnamese translation. Lastly, several items were added in our adapted version, including: “produced in a humane way”, “is not forbidden in my religion”, and “safe” (level of additional chemistry is within an acceptable level, kept clean, no bacteria”).

Socio-demographic variables: These variables were extracted from the consumer behavior survey, including gender, age, household size, and education level of the household representative.

Nutrition knowledge score: This score was measured from 30 questions that cover many aspects of diet and nutrition. The nutrition knowledge score captures knowledge of micronutrients, under- or over-nutrition, and dietary diversity [20]. The response was rated 1 as correct, or otherwise as 0. We then calculated the proportion of correct responses.

### 2.3. Diet Quality Indices

We assessed diet quality using the Diet Quality Index–International (DQI-I) [21] and in Vietnamese context, namely DQI-V. This is a combination of nutrient- and food/food group-based diet quality indicators and provides a more comprehensive picture of diet quality than nutrient or food/food groups alone. The DQI-V is composed of four components representing four aspects of a diet: variety, adequacy, moderation, and balance, each of which has subcomponents. The construction method of DQI-V was as follows: scores of each of the four components were calculated separately, then the total DQI-V was calculated as the sum of these component scores, resulting in a total score between 0 and 100: 0 being the poorest, indicating a diet of the lowest quality, and 100 being the best, indicating a diet of the highest quality. The DQI-V scoring procedure was adapted to Vietnamese dietary guidelines including the Recommended Dietary Guidelines [16] and the Nutritional Pyramid for Vietnamese people [22]. The detailed scoring system of the DQI-V and its four components are described in Table S1 in the Supplementary Materials.

### 2.4. Empirical Model

We conducted an exploratory factor analysis (EFA) to define the latent variables. These latent variables (or factors) are used to indirectly measure complex concepts that cannot be directly observed (here, referring to the underlying dimensions of food choice motives). The latent variables are constructed from the FCQ. Firstly, we made several tests and measures to determine how suited FCQ data is for factor analysis.

The Kaiser–Meyer–Olkin (KMO) Test measures sampling adequacy, based on the proportion of variance among variables, for each variable in the model, and the complete model. The reference values of the KMO test, which vary between 0 and 1, relative to the adequacy of the factor analysis, are as follows: between 0.900 and 1.000–very good; between 0.800 and 0.900–good; between 0.700 and 0.800–average; between 0.600 and 0.700–reasonable; between 0.500 and 0.600–bad; and less than 0.500–unacceptable.

Bartlett’s Sphericity test measures whether the samples have equal variances (called homogeneity of variances) and is based on the Chi-squared statistical distribution. This test is vital due to the homogeneity of variances assumption in the factor analysis, which is based on the analysis of variance. This test’s significance levels, or *p*-value, must be less than 0.05.

Cronbach’s alpha test is a measure of internal consistency reliability that ranges from 0 to 1. The internal consistency is greater the closer the statistical value is to 1. Cronbach’s alpha is classified as having appropriate reliability when the value is at least 0.70.

The EFA method is based on principal analysis with Promax rotation that allows for correlation among latent variables. We report factor loadings of each variable corresponding to their latent variables (or factors). According to Hair [23], factor loadings are the correlation of each variable, and they indicate the degree of correspondence between the variables. Factors with higher loadings make the variable representative of the factor. Each variable has loadings on all factors. In practice, factor loadings must be greater than 0.3. In detail, values ranging from 0.3 to 0.4 are considered as meeting the minimum level required for meaningful structure interpretation. Loadings 0.5 or greater are considered practically significant. Loadings exceeding 0.7 are considered indicative of well-defined structure and are the goal of any factor analysis.

Followed by a standard method as used by Hair [23], we performed a confirmatory factor analysis (CFA) based on the latent variables. This method showed the validity, reliability and the goodness-of-fit of the food choice motives being studied. There are various statistical indices of the CFA method, and we focus on the most popular indices, including Chi-square/degrees-of-freedom (χ2/df), GFI (goodness-of-fit index), CFI (comparative fit index), TLI (Tucker–Lewis index) and RMSEA (root mean square error of approximation). The reference values of these indices are in Table S3 in the Supplementary Materials.

The relationships between diet quality and food choice motive were analyzed by structural equation modeling (SEM) [24,25]. The SEM model can simultaneously examine a series of interrelated, dependent relationships which are therefore similar to multiple-regression equations. These equations depict all relationships among constructs (the dependent and independent variables) and variables involved in the analysis. Constructs are unobservable, or latent factors, represented by multiple variables.

## 3. Results

### 3.1. Descriptive Statistics

Table 1 presents the descriptive statistics concerning the socio-demographic variables of the sample. The shares of respondents living in rural and urban sites are comparable, while the number of households in the peri-urban site is slightly smaller. The proportion of males and females is similar. The majority (84%) of adults are at least high-school graduates. On average, households have five members and age of respondents is 31.3 years, regardless of site. The mean nutrition knowledge score for all households is 0.61 out of 1.0. The households earning less than VND 7 million (approximately USD 300) per month account for 34.7%.

**Figure 1:**
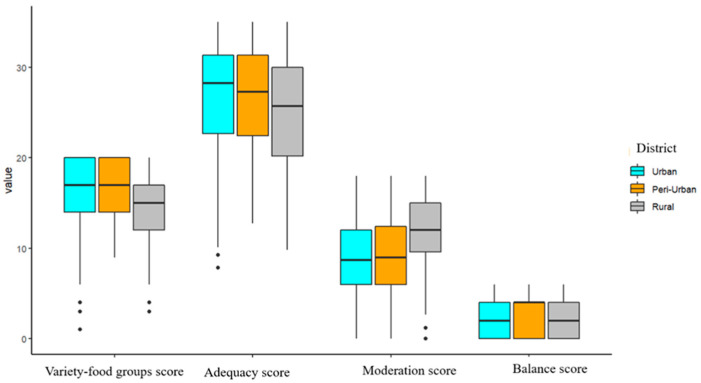
Boxplot of DQI-V by area.

The diet quality scores are reported as mean and standard deviation in Table 1 and each of the sites is depicted in Figure 1. The overall DQI-V score on average is 54.15 over 100, whereas the variety score (respectively, adequacy, moderation, and balance score) contributes 15.52 over 20 (respectively, 25.88 over 40, 10.21 over 30, and 2.54 over 10).

Figure 1 shows that the sizes of the boxplots of the four categories for three sites are almost the same. The rural site has a lower value for variety-food groups and adequacy score, but not for moderation score. Interestingly, 25% of urban and peri-urban sites obtain the maximum score for a variety of food groups, whereas 25% of all three areas have the lowest balance score. More descriptive statistics between gender, age, household size, education level, and income level are presented in the Supplementary Materials. The correlation between diet quality and household size and age are weak and not significant (Table S2 Supplementary Materials). The variations of diet quality by income level are similar, except for households having less than VND 7 million (as shown in the interquartile range of the boxplot) (see Figure S1—Supplementary Materials). By education level, households with higher education levels tend to score higher for variety-food groups score, adequacy score, and VQI-V score (Figure S2). However, an inverse trend for moderation score by education level is detected. Diet quality shows similar statistical values by male and female (Figure S3).

### 3.2. Exploratory Factor Analysis (EFA)

The results from the exploratory factor analysis (EFA) are presented in Table 2. Instead of nine factors as in the original hypothesis [1] (including health, mood, convenience, sensory appeal, natural content, price, weight control, familiarity, and ethical concern), the results show that they grouped into five factors. We labeled them as health focus, sensory appeal, mood and ethics, convenience, and familiarity. In addition, while four of these factors were mixed, sensory appeal stayed the same. For example, health focus was composed of health, natural, and weight control. The mood and ethic concern factors were combined. The convenience factor has two instead of three items. Finally, familiarity comprised two original items and one from the ethical concerns factor.

The Kaiser–Meyer–Olkin measure of sampling adequacy (KMO = 0.79 > 0.7) and *p*-value < 0.000 of the Bartlett’s test indicated the validity of the food choice dimensions in our study. The Cronbach’s alpha values of all dimensions are higher than 0.75 except for familiarity. These Cronbach’s alpha values are considered good as a reference [26]. The eigenvalues of the five factors explained 47% of the variance.

### 3.3. Confirmatory Factor Analysis (CFA)

We conducted a confirmatory factor analysis (CFA) on the five-factor, 22-item FCQ model resulting from the EFA. The CFA test shows how FCQ items and factors fit the observed data. We conducted multiple procedures to test the model fit to be able to make improvements to the model to find the best goodness-of-fit. These indicators ensure a good fit of the data with the research model [23,26]. We added some constraints among items in each factor, using residuals from the regression model and the modification indices (=univariate score tests). According to the CFA results, the main indicators of the model’s fit are χ2/df = 3.988 < 5; GFI = 0.91 > 0.9; CFI = 0.897 > 0.85; TLI = 0.862, and RMSEA = 0.070 < 0.1. All indices show the model fit at an acceptable level as required [26].

Based on the standardized regression indexes and the correlation coefficients of the variables from the CFA’s results, we calculated the composite reliability (CR) and the composite reliability indexes. The convergent validity and discriminant validity of the variables are shown in Table 3. Following [26], the scale meets the requirements of general reliability when CR > 0.7, except familiarity = 0.62; meets the requirements of convergence value when the average extracted variance AVE > 0.3, and achieves discriminant value when AVE > MSV and the square root of the AVE of each variable is greater than the correlation coefficient between that variable and other variables in the model. As shown in Table 4, the variables of the research model mainly satisfy the above requirements, except for familiarity and convenience.

### 3.4. Structural Equation Model

The structural equation model (SEM) was used to find the relationship between food choice motives and diet quality, together with socio-demographic characteristics of observations. Using five diet indicators: variety-food groups score, adequacy score, moderation score, balance score, and DQI-V, we first ran a total of five SEM models. For each diet indicator as a dependent variable, we ran two analytical models with and without control variables. We used six control variables: districts; education; gender; nutrition knowledge score; income (millions of VND); and age (years). Models with control variables are sufficiently robust to determine the influence of the main variables, given the characteristics of the data. Details of the estimating SEMs are presented in Table 4 and Table 5. In both tables, the important statistical indicators of fit (model fit), including GFI, CFI, RMSEA, and χ2/df, showed that the analytical models were in strong agreement with the collected data [23,26].

In Table 4, sensory appeal, convenience, and familiarity variables had a statistically significant association on the DQI-V score. All independent variables have no effect on moderation score and balance score, respectively. The mood-ethics factor and convenience factor significantly impacted the adequacy score. In addition, health focus, sensory appeal, and convenience factors played important roles in explaining the variety-food groups score.

However, when adding control variables to the analytical models, the results had some changes (Table 5). The sensory appeal variable no longer had a statistically significant association while the mood-ethics factor had a positive association with the DQI-V score. The convenience factor negatively affected the variety-food group’s score while the other four factors did not have any statistically significant association. Some results were the same as in Table 4. For example, there was no impact of all control variables on the dependent variables of moderation and balance scores. The mood-ethics and convenience factors had statistically significant associations with the adequacy score.

In addition, in terms of control variables, district areas had a statistically significant negative association with the variety-food groups scores but not with other scores. Education level also had adverse effects on moderation scores. Gender had a statistically significant positive association with the DQI-V score and adequacy score. The nutrition knowledge had a statistically significant positive association with all scores, except it negatively affected the moderation score. Income substantially affected the moderation scores and variety-food groups scores. Finally, age had a negative influence on adequacy scores.

## 4. Discussion

This study used both EFA and CFA to identify the factor groupings that best encompass Vietnamese consumers’ motives for food choice. Using EFA, we found that a grouping of five factors instead of the original nine factors can better reflect the perceptions of respondents on food choices. These five contextually appropriate food choice motive factors are: health focus, sensory appeal, mood-ethics, convenience, and familiarity. In addition, the modification index helped to connect items in the same latent variables to improve the model fit.

The confirmation of five core consumer motives of Vietnamese households along the rural–urban transect has a significant implication for nutrition programs in the country. The importance of these five core motives is well established. Health-associated properties of food have typically been a key driver for Vietnamese consumers’ choices [27,28]. This is in line with another recent qualitative study conducted at the same three sites, indicating that safe and nutritious food for better health and weight gain was in focus by mothers when selecting food to feed their children [22]. Although the status of consumer knowledge about micro- and macronutrient content is not clear, there is a strong perception of health properties related to foods and how these were produced.

Vietnamese sensory food preferences regarding appearance, smell, texture, and taste are well differentiated and unique within Southeast Asia. Popular cuisine typically includes fresh vegetables, herbs, rice and noodles, fish, and meats, with limited amounts of dairy, and oil or processed animal products. Contrasts or the ‘yin and yang’ are essential for composing what Vietnamese would consider a sensory balanced meal. In essence, there is an as yet scientifically unrevealed set of characteristics that culturally differentiates the sensory contours of Vietnamese consumers.

Eating food for social bonding, celebrations, and to define one’s mood is a vital part of Vietnamese food culture. Ethical and environmental concerns about foodborne diseases and agrochemical residues are deeply rooted in more recent concerns about ultra-processed and energy-dense foods [29,30,31]. Convenience on the other hand, is likely a criterion that has evolved as rapid economic development in the country has imposed time constraints on daily household activities such as food preparation. Women are a highly active workforce in Vietnam [32]. Yet culturally, women are a still expected to take care of food preparation, thus leading to time constrains for young (peri-)urban families who are increasingly living independently. Familiarity is a strong, yet sparsely researched, factor underlying Vietnamese food choices. Its importance can be indirectly observed from the comparatively modest presence of foreign outlets in the Vietnamese food environment compared to other Southeast Asian countries.

The results yielded from the factor analysis in the initial food choice questionnaire were in line with the results of several previous studies. For example, Pula et al. [33] showed that in a sample of US residents, some original FCQ factors such as “weight control” and “ethical concern” were either eliminated or modified, while a new “impression management” factor was included. Fotopoulos et al. [34] concluded that FCQ had too many dimensions with high levels of abstraction, and would benefit from fewer dimensions or fewer items per dimension. In the Vietnamese contexts, “weight control” can be combined with health as people generally associate adequate weight with good health. The dimension “natural content” is also best clustered with “health focus”, as natural ingredients, such as oriental herbs and spices, have been integral to the Vietnamese culture and valued as beneficial for health for having medicinal properties. Mood and ethics can be combined in the Vietnamese contexts, as these aspects of food choice are both considered sentimental.

Our findings also reinstated the findings of a systematic review by Cunha et al. [8], stating that the FCQ is “neither comprehensive nor complete enough to be used in all scopes”. Methodologically, the questionnaire should be adapted to suit local concerns. For example, additional factors/dimensions and associated items can be included to reflect the cultures. Most Buddhists in Vietnam do not eat meat and such religious reasons should be included and tested. In the Asian population, the “religion” dimension was frequently included in the measurement of the motives of consumption, standing out as one of the most-valued factors [6]. Another relevant dimension is traditional norms and wisdom, which has been found to dictate Vietnamese dietary practices, even after migrating to other countries [34].

The SEM results indicated that food choice motives significantly impacted the DQI-V and its components, but in different directions. Sensory appeal had a positive effect on the overall DQI-V score while having a negative impact on the variety-food groups score. This finding may pose a potential dilemma for interventions and policies concerning food products: the opposite effects of sensory appeal (i.e., food should look good) and variety (i.e., a wide variety of sources should be offered) on diet quality.

We also demonstrated the use of DQI-V by exploring its associations with socio-economic characteristics. We found that the nutrition knowledge score is positively associated with all elements of diet quality, across all three sites. This result, together with the above findings, highlights the importance of improving nutrition knowledge for making better-informed food choices. Similarly, healthy weight, body mass index range, and health outcome of food to improve diet quality through interventions should be prioritized when working directly with women, caregivers, food service providers (especially people purchasing and/or preparing food in collective kitchens), or change agents such as teachers and health staff [25].

Being a rural resident showed a statistically significant negative association with only the variety-food group score, but not the other components. To a large extent, this is related to rural residents’ higher dependence on home-produced food and lower income [35] and purchasing power, compared to peri-urban and urban residents. Northern Vietnam’s high agrobiodiversity levels could be a tool to increase dietary diversity [35].

Differentiated policies are instrumental in addressing the poor dietary diversity and adequacy prevalent in northern Vietnam. Rising overconsumption of unhealthy foods in urban areas, for example, requires a tailored approach which the government is already mainstreaming [14,36,37,38]. One option involves labeling to provide information to consumers to help them identify healthier food choices.

This study provided more evidence concerning food choice motives in the food culture of Asian countries. Food choice questionnaires have been tested in several countries, including Japan, Taiwan, Malaysia, and New Zealand. Prescott et al. confirmed nine factors of food choice motives [39]. When comparing across countries, our results were in line with New Zealand consumers on the importance of sensory appeal. In addition, Vietnamese consumers and Japanese consumers expressed ethical concerns. Prescott et al.’s results also indicated that Taiwanese and (ethnically Chinese) Malaysian consumers pay more attention to natural content, weight control, but not in our study in Vietnam. Another study showed that Chinese people have some similar food choice motives as Vietnamese people where they also paid attention to sensory appeal, familiarity, and mood motives [39]. In term of other characteristics, empirical studies in Asian countries showed different trends. One study confirmed that there is no difference in food choice motives for Japanese people. Another study in China showed that there was a difference between types of food choice motives and well-being among young and middle-aged Chinese adults.

## 5. Conclusions

In this paper, we use EFA and CFA to validate the five factors on food choice motives based on the original nine factors for Vietnam—a low to middle-income country. Key factors for the Vietnamese context include health focus, sensory appeal, mood-ethics, convenience, and familiarity. This study reveals the importance of these motives for the first time for the Vietnamese context. It also aimed to link food choice motives (health focus, sensory appeal, mood-ethics, convenience, and familiarity) with diet quality, namely DQI-V score. The DQI-V has four major aspects of a high-quality diet, i.e., variety, adequacy, moderation, and overall balance, and is constructed based on the Vietnamese recommended dietary guidelines [21]. According to the range of DQI-V scores and its components, average scores of Vietnamese diets are around half of the maximum value, while variety obtained around 25% of maximum score, but the other three components (adequacy, moderation, and overall balance) can be improved significantly.

The relationship between DQI-V and five factors of the FCQ was explored using the SEM model. The results of the SEM analysis revealed that food choice motives had a significant impact on DQI-V and its components, but the effects were different. Specifically, sensory appeal had a positive impact on the overall DQI-V score but negatively affected the variety of food groups score. This finding presents a potential trade-off issue for interventions and policies related to food products as sensory appeal and variety have opposite effects on diet quality.

Additionally, the study explored the relationship between DQI-V and socio-economic characteristics. The results showed that nutrition knowledge was positively associated with all elements of diet quality across all three study sites. This highlights the importance of improving knowledge of nutrition to enhance diet quality.

The study also found that being a rural resident had a negative association with only the variety-food group score, which was related to the higher dependence of rural residents on home-produced food and lower income and purchasing power compared to peri-urban and urban residents. The high levels of agrobiodiversity in northern Vietnam could be utilized to increase dietary diversity. Differentiated policies are necessary to address the poor dietary diversity and adequacy in northern Vietnam.

This study has some limitations. The results could have been more representative for Vietnamese consumers if we had been able to include more participants from other provinces in Vietnam in the survey. In addition, the link between food choice motives and diet quality would show more comprehensive results if we included nutrition outcomes, such as body mass index or well-being variables in our analysis.

## Figures and Tables

**Table 1:** Description of socio-demographic variables.

Variables		Values
Number of observations, n		603
Household size		5.07 (1.40)
Districts	Peri-urban (%)	27.86
	Rural (%)	36.48
	Urban (%)	35.66
Nutrition knowledge score		0.61 (0.19)
Age (years)		31.33 (6.42)
Income (millions of VND) (%)	Less than 7	34.66
	From 7 to 11	25.54
	From 11 to 20	19.73
	Greater than 20	20.07
Education levels (%)	Primary school or no formal education	16.09
	Secondary school	20.56
	High school	21.39
	University and college	41.96
Gender (%)	Male	50.08
	Female	49.92
Variety-food groups score		15.52 (3.85)
Adequacy score		25.88 (6.15)
Moderation score		10.21 (4.60)
Balance score		2.54 (2.16)
Diet Quality Index–International score		54.15 (8.98)

Continuous variables are reported as mean (standard deviation) and categorical variables are reported as percentage.

**Table 2:** The scales, items, Cronbach’s alpha, and loading factors.

Latent Variable	Item		Cronbach’s Alpha	Loading Factor
	Code	Description		
Health focus			0.85 *	
	H1	Is high in vitamins and minerals	0.81	0.86
	H4	Is high in protein	0.82	0.79
	H6	Is high in fiber and roughage	0.81	0.82
	NC1	Contains no additives	0.84	0.53
	NC3	Contains natural ingredients and little or no artificial ingredients	0.84	0.45
	WC1	Is low in calories	0.84	0.57
	WC2	Is low in fat	0.83	0.67
Sensory appeal			0.79 *	
	SA1	Smells nice	0.67	0.95
	SA2	Looks nice	0.72	0.77
	SA3	Has a pleasant texture	0.8	0.54
	SA4	Tastes good	0.77	0.64
Mood and Ethics			0.76 *	
	M1	Makes me feel good emotionally (cheers me up, helps me cope with stress, relax, etc.)	0.71	0.63
	M2	Keeps me awake/alert	0.69	0.79
	NC2	Is produced without chemicals (e.g., pesticides)	0.77	0.4
	EC3	Is packaged in an environmentally friendly way	0.68	0.8
	EC4	Produced in a humane way	0.72	0.58
Convenience			0.8 *	
	C4	Can be bought in shops close to where I live or work	0.66	0.87
	C5	Is easily available in shops and supermarkets	0.66	0.67
Familiarity			0.61 *	
	F2	Is advertised in the media (television, radio, internet, etc.)	0.51	0.51
	F3	Is recommended by my friends or other people who are important to me	0.53	0.45
	EC1	Comes from countries I approve of politically	0.61	0.37
	EC5	Is not forbidden in my religion	0.53	0.66

Note: The EFA analysis is based on the principal analysis with Promax rotation. Seven items have been eliminated because they do not have loading factors greater than 0.37. Cronbach’s alpha coefficients with a star (*) are the alpha of the latent variables.

**Table 3:** Measurement of the reliability, convergent validity, and discriminant validity of the five-factor model of food choice motives.

Factors	CR	AVE	MSV	SQRT (AVE)			
				Health Focus	Sensory Appeal	Mood-Ethics	Convenience
**Health focus**	0.85	0.45	0.42				
**Sensory appeal**	0.82	0.56	0.3	0.2 ***			
**Mood-Ethics**	0.72	0.34	0.42	0.65 ***	0.3 ***		
**Convenience**	0.8	0.68	0.17	0.33 ***	0.24 ***	0.41 ***	
**Familiarity**	0.62	0.29	0.13	0.29 ***	0.17 ***	0.26 ***	0.36 ***

Note: CR: composite reliability; AVE: average variance extracted; MSV: maximum shared variance; SQRT (AVE): square root of average variance extracted. *p*-value: *** < 0.001.

**Table 4:** Structural equation models assessing the impact on Diet Quality Index–International score and its components.

Variables	DQI-V Score	Moderation Score	Balance Score	Adequacy Score	Variety-Food Groups Score
**Health focus**	0.67 (0.28)	−0.5 (0.13)	0.92 (0.11)	0.55 (0.2)	0.54 * (0.05)
**Sensory appeal**	−1.12 * (0.05)	0.14 (0.63)	0.88 (−0.09)	−0.57 (0.13)	−0.58 * (0.02)
**Mood-Ethics**	2.08 (0.07)	−0.31 (0.61)	0.65 (0.09)	1.7 * (0.03)	0.59 (0.24)
**Convenience**	−2.54 *** (0)	0.43 (0.25)	0.19 (−0.21)	−1.97 *** (0)	−0.77 * (0.01)
**Familiarity**	1.16 * (0.05)	0.34 (0.23)	0.8 (0.01)	0.61 (0.12)	0.15 (0.54)
**Adjusted** $R^{2}$	0.05	0.01	0.01	0.05	0.05
**Model fit**					
GFI	0.91	0.92	0.92	0.91	0.91
CFI	0.9	0.91	0.91	0.9	0.9
RMSEA	0.06	0.06	0.06	0.06	0.06
χ2/df	3.49	3.39	3.33	3.5	3.53

Note: *p*-value: * < 0.05, *** < 0.001.

**Table 5:** Structural equation models assessing the impact on the Diet Quality Index–International score and its components.

Variables	DQI-V Score	Moderation Score	Balance Score	Adequacy Score	Variety-Food Groups Score
**Health focus**	0.22 (0.72)	0.24 (0.42)	−0.01 (0.92)	0.00 (1.00)	−0.02 (0.93)
**Sensory appeal**	−0.98 (0.07)	−0.13 (0.65)	−0.02 (0.88)	−0.46 (0.22)	−0.36 (0.11)
**Mood-Ethics**	2.26 * (0.05)	−0.58 (0.3)	0.13 (0.65)	1.9 * (0.01)	0.82 (0.08)
**Convenience**	−2.45 *** (0)	0.5 (0.16)	−0.23 (0.19)	−1.9 *** (0)	−0.82 ** (0.01)
**Familiarity**	1.18 * (0.04)	0.06 (0.84)	0.03 (0.8)	0.76 (0.05)	0.31 (0.19)
**Districts**	−0.68 (0.15)	0.09 (0.72)	−0.2 (0.09)	−0.12 (0.72)	−0.45 * (0.02)
**Education**	−0.59 (0.07)	−0.64 *** (0)	−0.01 (0.87)	−0.06 (0.79)	0.13 (0.34)
**Gender**	1.56 * (0.03)	0.08 (0.82)	−0.05 (0.8)	1.68 *** (0)	−0.16 (0.6)
**Nutrition knowledge score**	6.73 *** (0)	−4.8 *** (0)	1.53 ** (0)	5.26 *** (0)	4.75 *** (0)
**Income (millions of VND)**	−0.57 (0.09)	0.33 * (0.05)	−0.09 (0.26)	−0.36 (0.11)	−0.44 ** (0)
**Age (years)**	−0.07 (0.25)	0.04 (0.17)	0.00 (0.9)	−0.09 * (0.03)	−0.02 (0.33)
**Adjusted** $R^{2}$	0.1	0.1	0.03	0.1	0.12
**Model fit**					
GFI	0.87	0.87	0.87	0.87	0.87
CFI	0.86	0.87	0.87	0.86	0.86
RMSEA	0.06	0.06	0.06	0.06	0.06
χ2/df	3.24	3.15	3.14	3.23	3.23

Note: *p*-value: * < 0.05, ** < 0.01, *** < 0.001.

## Data Availability

The data used to support the findings of this study can be made available by the corresponding author upon request.

## Supplementary Materials

The following supporting information can be downloaded at: https://www.mdpi.com/article/10.3390/foods12132505/s1, Table S1: Components of Vietnam Diet Quality Index–International (DQI-V). Table S2: Correlation coefficients between diet quality and household size and age. Table S3: Reference values of the quality indicators for a confirmatory factor analysis. Figure S1: Boxplot of DQI-V by income levels. Figure S2: Boxplot of DQI-V by education levels. Figure S3: Boxplot of DQI-V by gender.
