# Supplementary material for: Diet Quality Index and Food Choice Motives in Vietnam: The Roles of Sensory Appeal, Mood, Convenience, and Familiarity
Source: Foods. 2023 Jun 28;12(13):2505. doi: 10.3390/foods12132505 (PMC10341352; doi:10.3390/foods12132505)
Supplement: Supplementary file 1 [file foods-12-02505-s001.zip › foods-2381382-supplementary.pdf]

Supplementary Materials

Table S1: Components of Vietnam Diet Quality Index-International (DQI-V)

Component	Score	Scoring criteria	Detail and meaning
**Variety**	**0–20 points**		Variety was evaluated in two ways: overall dietary variety, and variety within protein sources, to assess whether intake comes from a wide variety of sources both across and within food groups. Beans and dairy are combined into the same food group to serve the DQI-V's intended use in cross-national comparisons. Since beans are a major source of calcium in many developing countries in, intake of dairy foods alone would considerably underrepresent the actual intake of calcium-rich foods in developing countries (S. Kim et al. 2003). Variety among the protein sources (meat, poultry, fish, dairy, beans, and eggs) is incorporated in the Variety component to illustrate the benefits of including diverse food sources in the diet even within the same food group. Intake of more than half the serving size per day from each of the above protein sources is considered to be meaningful consumption. When intake is derived from at least three different sources of protein per day, the maximum score was given. The scores from the overall variety and variety within protein sources were then summed to get the total Variety scores.
Overall food group variety (meat/poultry/fish/eggs; dairy/beans; grain; fruit; vegetable)	0–15 points	>= 1 serving from each food group/d = 15	
		Any 1 food group missing/d = 12	
		Any 2 food groups missing/d = 9	
		Any 3 food groups missing/d = 6	
		Any 4 food groups missing/d = 3	
		None from any food group = 0	
Within-group variety for protein source (meat, poultry, fish, dairy, beans, eggs)	0–5 points	>=3 different sources/day = 5	
		2 different sources/d = 3	
		From 1 source/d = 1	
		None = 0	
**Adequacy**	**0–40 points**		This component assesses the intake of dietary elements that must be supplied adequately to avoid undernutrition and deficiency disorders. The subcomponents of Adequacy are vegetables, fruits, grains, fiber, protein, iron, calcium, and vitamin C.
Vegetables[a]	0–5 points	>=4 servings/d = 5, 0 servings/d = 0	
		>= 100% RDA	
		< 100-50%	

Component	Score	Scoring criteria	Detail and meaning
		<50%	The scores for the eight mentioned subcomponents are assigned based on the percentage attainment of the recommended intakes from the Vietnamese dietary guidelines on a continuous scale, which ranges from 0 points for 0% to 5 points for 100%, with a cap at 5 points. Specifically, we first calculated the percentage attainment of the recommended intake for each subcomponent, then based on that percentage we derived the point where 0 points for 0%, 5 points for 100%, and the percentage attainment in between was calculated proportionately.
Fruits[a]	0–5 points	>=3 servings/d = 5, 0 servings/d = 0	
		>= 100% RDA	
		< 100-50%	
		<50%	
Grains[a]	0–5 points	>= 12 servings/d = 5, 0 servings/d = 0	
		>= 100% RDA	
		< 100-50%	
		<50%	
Fiber[a]	0–5 points	***Men:***<br>15 - 19 years old: >= 38 g/d<br>20 - 29 years old: >= 38 g/d<br>30 - 49 years old: >= 38 g/d<br>50 - 69 years old: >= 30 g/d<br>***Women:***<br>15 - 19 years old: >= 26 g/d<br>20 - 29 years old: >= 25 g/d<br>30 - 49 years old: >= 25 g/d<br>50 - 69 years old: >= 21 g/d	
		>= 100% RDA	
		< 100-50%	
		<50%	
Protein[a]	0–5 points	>= 13 - 20% of total energy/d = 5, 0% of energy/d = 0	
		>= 100% RDA	
		< 100-50%	
		<50%	

Component	Score	Scoring criteria	Detail and meaning
Iron[a, b]	0–5 points	***Men***<br>15 - 19 years old: >= 11.6 mg/d<br>20 - 29 years old: >= 7.9 mg/d<br>30 - 49 years old: >= 7.9 mg/d<br>50 - 69 years old: >= 7.9 mg/d<br>***Women: Bioavailability 10%***<br>15 - 19 years old: >= 29.7 mg/d<br>20 - 29 years old: >= 26.1 mg/d<br>30 - 49 years old: >= 26.1 mg/d<br>50 - 69 years old: >= 26.1 mg/d	
		>= 100% RDA	
		< 100-50%	
		<50%	
Calcium[a, b]	0–5 points	***Men:***<br>15 - 19 years old: >= 1000 mg/d (RDA), <3000 mg/d (UL)<br>20 - 29 years old: >= 800 mg/d, <2500 mg/d<br>30 - 49 years old: >= 800 mg/d, <2500 mg/d<br>50 - 69 years old:>= 800 mg/d, <2000 mg/d<br>***Women:***<br>15 - 19 years old: >= 1000 mg/d (RDA), <3000 mg/d (UL)<br>20 - 29 years old: >= 800 mg/d, <2500 mg/d<br>30 - 49 years old: >= 800 mg/d, <2500 mg/d<br>50 - 69 years old :>= 900 mg/d, <2000 mg/d	

Component	Score	Scoring criteria	Detail and meaning
		>= 100% RDA	
		< 100-50%	
		<50%	
Vitamin C[a, b]	0–5 points	>=100 mg/d = 5, 0 mg/d = 0	
		>= 100% RDA	
		< 100-50%	
		<50%	
**Moderation**	**0–30 points**		Moderation evaluates the intake of food and nutrients that are related to non-communicable diseases, and therefore may need restricted intake (S. Kim et al. 2003). The subcomponents of Moderation are total fat, saturated fat, cholesterol, sodium, and empty calorie food (S. Kim et al. 2003). Except for empty calorie food, the other subcomponents were modified to follow the guidelines from the VDG. Empty calorie foods are foods that provide energy but insufficient nutrients (S. Kim et al. 2003). This study pre-defined empty calorie foods as the following food items which contain solid fats and added sugars: bacon, cakes, cheese, cookies, energy drinks, ice cream, fruit drinks, hot dogs, pastries, pizza, sodas, sports drinks, sausages and ribs (United States Department of Agriculture 2015). The intake levels of the subcomponents of Moderation are categorized into three tiers, according to the degree of effect on the health of a normal person (S. Kim et al. 2003). Those in the lowest tier are where intakes show limited evidence of harmful effects (S. Kim et al. 2003). The highest tier is the boundary where overconsumption may be associated with chronic
Total fat	0–6 points	<= 20% total energy/d = 6	
		> 20-30% of total energy/d = 3	
		>30% of total energy/d = 0	
Saturated fat	0–6 points	<= 7% total energy = 6	
		> 7-10% total energy = 3	
		> 10% total energy = 0	
Cholesterol	0–6 points	<= 300 mg/d = 6	
		> 300-400 mg/d = 3	
		> 400 mg/d = 0	
Sodium	0–6 points	<= 15%tile (470mg/d) = 6	
		> 15%tile & <85%tile: proportionate	
		>= 85%tile (2090mg/d) = 0	
Empty calorie food	0–6 points	<=3% of total energy/d = 6	
		> 3-10% of total energy/d = 3	
		>10% of total energy/d = 0	

Component	Score	Scoring criteria	Detail and meaning
			health outcomes (S. Kim et al. 2003). The middle tier covers intakes between the lowest and highest tiers (S. Kim et al. 2003). The lowest tier category is given the highest score of 6 points, the highest intake category is given the lowest score of 0, and the middle tier is worth a score of 3 (S. Kim et al. 2003).
**Overall balance**	**0–10 points**		This category investigates the overall balance of a diet in terms of the proportion of energy sources from macronutrients (carbohydrate – protein – fat) and fatty acid composition (saturated fat – monounsaturated fat – polyunsaturated fat) (S. Kim et al. 2003). This section has been modified to observe the Vietnam Dietary Guidelines (VDG) (for nutrients).<br>Serving size conversion: The original DQI-I uses the U.S. Food Guide Pyramid serving size definition (Food Surveys Research Group - Beltsville Human Nutrition Research Center 1999), this study used the guidelines from the Vietnam Food Pyramid for Adults in the period 2016 - 2020 (Annex 2). The detailed adapted DQI-V and its corresponding scoring criteria are described in Annex 4. A higher DQI-V score indicates a higher-quality diet with better variety, adequacy, moderation, and overall balance.
Macronutrient ratio - Carbohydrate:Protein:Fat	0–6 points	55~ 65 : 10~15 : 15~25 = 6	
		52~68 : 9~16 : 13~27 = 4	
		50~70 : 8~17 : 12~30 = 2	
		Otherwise = 0	
Fatty acid ratio - PUFA:MUFA:SFA	0–4 points	P/S =1 ~1.5 and M/S =1 ~1.5 = 4	
		Else if P/S = 0.8 ~ 1.7 and M/S = 0.8 ~ 1.7 = 2	
		Otherwise = 0	

Table S2: Correlation coefficients between diet quality and household size and age

	Household size	Age (years)	Nutrition knowledge score
Diet Quality Index International score	0.01	-0.03	0.16
Variety-food groups score	0.02	-0.03	0.28
Adequacy score	-0.04	-0.05	0.19
Moderation score	0.04	0.02	-0.24
Balance score	0.01	0.00	0.14

Figure S1: Boxplot of DQI-V by income levels

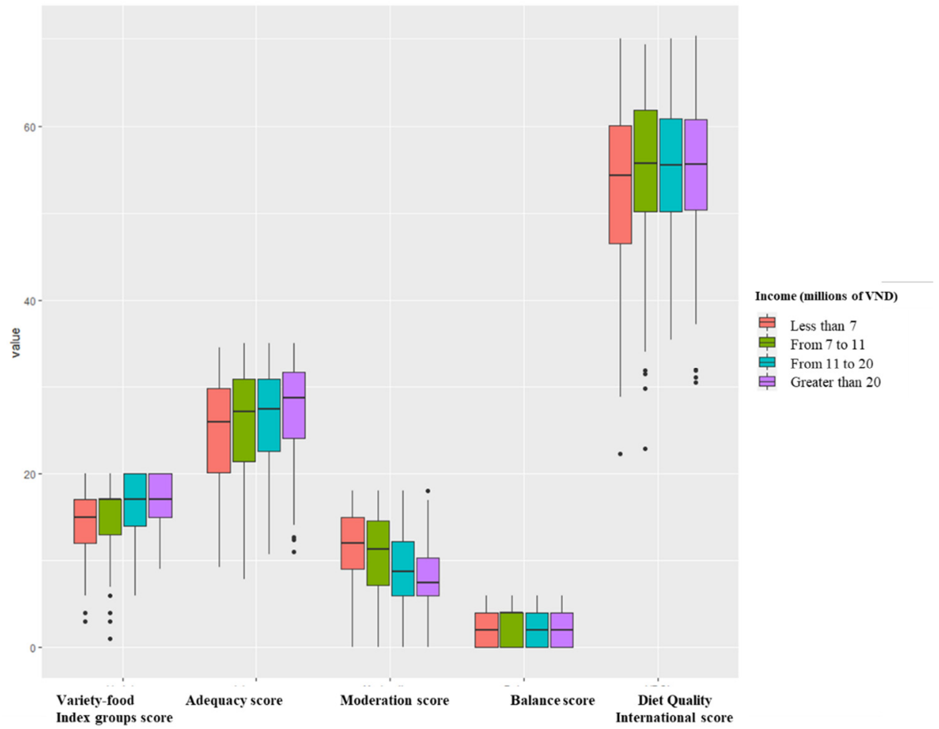

Figure S2: Boxplot of DQI-V by education levels

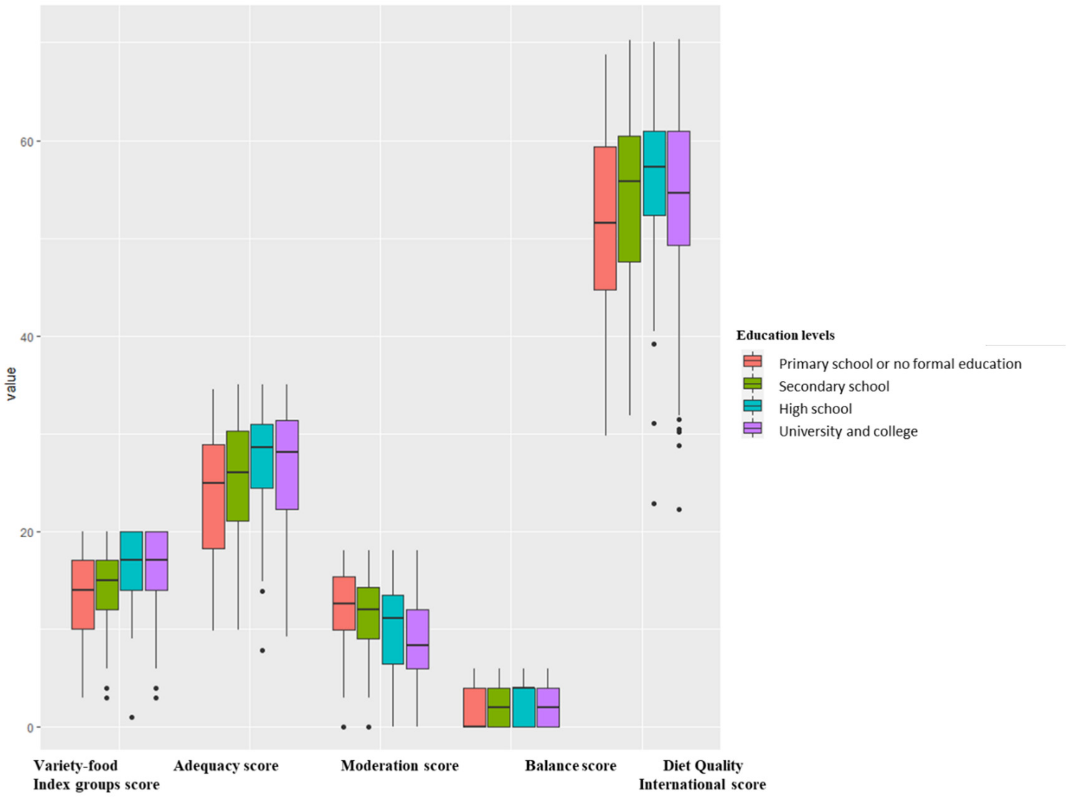

Figure S3: Boxplot of DQI-V by gender

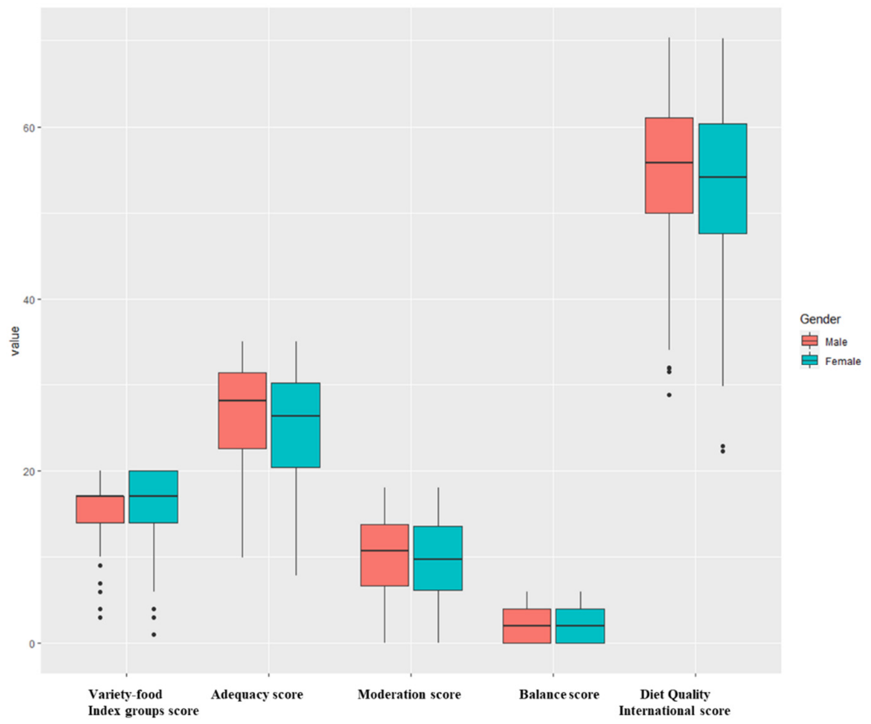

Table S3: Reference values of the quality indicators for a confirmatory factor analysis

Statistic Indices	Reference Values
Qui-square/degree-of-freedom ($\chi2$/df)	Greater than 5: Unacceptable fit<br>From 2 to 5: Acceptable fit<br>From 1 to 2: Good fit<br>Less than 1: Very good fit
GFI (Goodness-of-Fit Index),<br>CFI (Comparative Fit Index)<br>and<br>TLI (Tucker-Lewis Index)	Less than 0.8: Unacceptable fit<br>From 0.8 to 0.9: Acceptable fit<br>From 0.9 to 0.95: Good fit<br>Greater than 0.95: Very good fit
RMSEA (Standardized Root Mean Square Residual)	Greater than 0.1: Unacceptable fit<br>From 0.05 to 0.1: Good fit<br>Less than 0.05: Very good fit
